# The Heterogeneity of High-Quality Economic Development in China’s Mining Cities: A Meta Frontier Function

**DOI:** 10.3390/ijerph19116374

**Published:** 2022-05-24

**Authors:** Wei Xu, Jiahui Yi, Jinhua Cheng

**Affiliations:** School of Economics and Management, China University of Geosciences, Wuhan 430074, China; 2201810262@cug.edu.cn (W.X.); anitayjh@gmail.com (J.Y.)

**Keywords:** mining city, high-quality economic development, input-output allocation efficiency, meta-frontier method

## Abstract

The transformation of mining cities and the realization of high-quality economic development are complicated processes. The objective existence of abundant resource factor endowment in mining cities does not mean that resource allocation is in the optimal state and can play the greatest role. The optimal allocation of factors for the high-quality economic development of mining cities is more important than the resource factors. The input–output allocation efficiency of high-quality economic development under the common frontier and group frontier of 99 mining cities in China from 2006 to 2019 is calculated by using the data envelopment analysis method and common frontier model, and the pure technical efficiency and scale efficiency are decomposed. The results show that (1) the comprehensive technical efficiency values under both common frontiers and group frontiers show that the factor allocation efficiency in the process of high-quality economic development of different mining cities shows obvious heterogeneity. (2) The growth of the input–output allocation efficiency of the high-quality economic development of mining cities has significant spatial convergence characteristics, but the convergence speed is different. (3) The high-quality development path of the mining city’s economy should not only focus on comprehensively improving the ability of resource element input and output allocation but also improve the group environment.

## 1. Introduction

The mineral resource industry has made and is currently an important contribution to the development of the national economy. Mining cities, which rise and develop due to the development of mineral resources, become the main body of mineral resource supply and is the resource production, transfer and reserve hub of China’s industrialization, directly providing raw materials for industrialization. China’s resource-based cities are numerous and widely distributed, with great historical contributions and have a prominent status. Since the founding of the People’s Republic of China, resource-based cities have produced 52.9 billion tons of raw coal, 5.5 billion tons of crude oil, 5.8 billion tons of iron ore, and 2 billion cubic meters of wood. During the First Five-Year Plan period, 53 of the 156 national key construction projects were distributed in resource-based cities, accounting for nearly 50% of the total investment, making historic contributions to the establishment of an independent and complete industrial system and the promotion of national economic development.

At present, international political and economic uncertainty and instability are rising, and the unbalanced, uncoordinated and unsustainable challenges in domestic economic development are prominent. Due to the superposition of internal and external factors and the interweaving of new and old contradictions, the sustainable development of mining resource-based cities faces severe challenges, and the task of accelerating the transformation of the economic development mode is very arduous [1]. The historical legacy of mining resource-exhausted cities is still serious, and the endogenous power of transformation and development is currently weak [1,2,3] Dan et al., 2019. There are still nearly 70 million square meters of shantytowns to be renovated, approximately 140,000 hectares of subsidence areas to be treated, more than 600,000 unemployed miners, and more than 1.8 million urban subsistence allowances. Industrial development is still highly dependent on resources, with extractive industries accounting for more than 20% of secondary industries, and modern manufacturing and high-tech industries are in the initial stages. The talent, capital and other elements of the mining resources city have weak agglomeration capacity and low allocation level such that the support and guarantee capacity for further development of alternative industries is seriously inadequate [4,5,6].

However, the level of technological progress and the quality of economic development represented by total factor productivity and technological efficiency do not show an obvious trend of growth or improvement. Some of the important reasons is that the resource allocation structure is not good, the efficiency is not high, and the overall efficiency of the national and regional urban resource allocation system is not high. In short, the input–output allocation of resource elements is a complicated process, especially for mining resource-based cities, and the input of resource elements is only a necessary condition. Whether the input of resource elements can improve the level of economic development and promote the high-quality development of the economy depends on whether the allocation of input resource elements can be optimized. The input–output allocation of high-quality economic development is a dynamic process of searching and obtaining, distributing and managing, integrating and utilizing, maintaining and updating various material or nonmaterial resource elements in a certain time and mining resource city by government, enterprises, colleges and universities, science and technology intermediary service institutions and nonprofit organizations. Therefore, it is of great significance to promote the sustainable development of mining resource-based cities to promote new industrialization and urbanization and to build a resource-saving and environmentally friendly society. Based on the perspective of the input–output efficiency of resource element allocation in mining cities in China, this paper intends to reveal the internal causes of the deviation of the input and output of resource elements in mining cities and the increasingly obvious and unbalanced spatial agglomeration.

## 2. Literature Review

With the deepening of resource-based cities, scholars have gradually realized the complexity of the economic growth of mining resource-based cities, and an increasing number of studies have also shown that the realization of mining resource-based cities needs to integrate and reuse the input–output resource elements in the process of economic growth [2,7,8,9,10].

With regard to the research on the input–output efficiency of high-quality economic development of mining resource-based cities in China, scholars have calculated and analyzed the effect or efficiency of input–output allocation with different evaluation indices and methods around different dimensions. Application of the statistical management method, Bui et al. (2020) [11] discusses the economic efficiency of the urban waste management system through the AHP-IPA method, Yuan et al. (2019) [12] discusses the spatial-temporal distribution characteristics of the land use efficiency of mining cities using the index decomposition method, and [13] studies the transition development level of Shaanxi, Shanxi and Inner Mongolia resource cities based on the structure decomposition model.

The most literature discuss the eco-environmental, energy and economic efficiency of resource-based cities through the stochastic frontier analysis model (SFA) and data envelopment method (DEA). Representative studies, such as Chen et al. (2019) [7], used the DEA method to study the industrial land use efficiency of 109 resource-based cities in China from 2006 to 2015. Yu et al. (2019) [14] discussed the ecological efficiency of resource-based cities under heterogeneous conditions through a DEA model, and Yin et al. (2020) [15] studied the green transformation efficiency of mining resource-dependent cities through a three-stage DEA model. In terms of econometric application, Yan et al. (2019) [2] used the nonparametric method to estimate the total factor energy efficiency (TFEE) of 105 resource-based cities in China from 2010 to 2016 and analyzed the temporal and spatial characteristics of the change energy efficiency.

The results show that the input–output efficiency of natural, environmental, ecological and transformation of mining resource-based cities is not ideal, and the development level difference between cities is gradually expanding. According to the level of factor allocation and its related influencing factors, the input and output indices of the economic development of mining resource-based cities are redundant. There is still room for further improvement in the existing relevant research, which is mainly reflected in the following aspects: first, the impact of resource input is ignored, and such output indicators are rarely involved; second, in terms of calculation methods, whether using the DEA or SFA method, it is assumed that all regions have the same technology set, obviously the technical gap between different types of mining cities is not considered, and the reason for efficiency loss cannot be determined. In view of this, on the one hand, the paper considers the impact of resource factor input on the input–output efficiency of high-quality economic development of mining resource-based cities and introduces the industrial agglomeration index into the measurement model of input–output efficiency of high-quality economic development of mining resource-based cities to avoid single factor resource input measurement; on the other hand, in view of the heterogeneity characteristics of different types of mining cities, the paper introduces the production function of the common frontier and comprehensively uses the meta-frontier model to calculate the input–output efficiency of high-quality economic development of mining resource-based cities under the common frontier and group frontier. This paper discusses the characteristics of the high-quality economic development of mining resource cities.

## 3. Research Design

### 3.1. Model

The requirements for high-quality economic development of China’s mining cities can generally be divided into five categories: human resources, financial resources, material resources, technology and information. The process of high-quality economic development of China’s mining cities is complex and malleable, and a single index cannot accurately measure it, while the efficiency of input–output allocation in the process of economic development of China’s mining cities can reflect the level of high-quality economic development to a certain extent. When the DEA method is used to measure the high-quality economic development of mining cities in different regions of China, the potential hypothesis is that the evaluated decision-making unit (DMU) has a similar technical level. However, with the existence of regional heterogeneity, it is impossible to accurately measure the allocation efficiency of the real high-quality economic development factors of each mining city by using the population sample only. Battese and Rao (2002) [16] and O’Donnell, Rao and Battese (2008) [17] proposed a common boundary production function analysis framework, using the stochastic frontier analysis method to construct a common frontier and group frontier, and finally measuring the technical gap ratio (TGR) between the two frontiers can make up the input–output efficiency under the condition that traditional DEA cannot measure heterogeneity. Common frontier approaches based on DEA are briefly described below [18,19].

(1) First, according to the National Sustainable Development Plan for Resource-based Cities (2013–2020) issued by the Chinese government, there are 262 resource-based cities in the country, including 126 prefecture-level administrative regions (these include prefecture-level cities, regions, autonomous prefectures, alliances, etc.), 62 county-level cities, 58 counties (including autonomous counties, forest regions, etc.), and 16 municipal districts (development zones, management zones). They are divided into four types: growth type (31), mature type (141), recession type (67) and regeneration type (23). Due to the large gap in resource endowment, factor input, output and allocation capacity of mature, regenerative, declining and growing mining cities and the large difference in macroeconomic level, mining city policy environment, opening level and other influencing factors, different types of mining cities face different production levels, and the internal difference of each type is smaller than the whole, so the research sample is divided into four groups: mature, regenerative, declining and growing four groups.

(2) According to the common frontier model of Battese and Rao (2002) [16], Zhang et al. (2013) [1], He et al. (2021) [20] let $input\in R^{m},output\in R^{n}$ is the *input* and *output* vector, and the common technology set (*TE^meta^*) including all *inputs* and *outputs* is:

*TE*^*meta*^ = {(*input*, *output*)|*input*, *output* ≥ 0; *input* → *output*} (1)

where *input* is the *input* vector and *output* is the *output* vector, which means the conditions that the required *input* satisfies under the technology set *TE^meta^* to obtain a certain *output product^meta^*. The set of production possibilities (common boundary) is:

*product*^*>meta*^ (*input*) = {*output*|(*input*, *output*)∈*TE*^*meta*^ } (2)

Common Frontier Distance Function (*DDF^meta^*) of Meta Technical Efficiency can be expressed as Formula (3), where 0 ≤ *DDF^meta^*(*input*, *output*) ≤ 1.

(3) $${\mathrm{DDF}}^{\mathrm{meta}}(\mathrm{input},\;\mathrm{output})=\inf_{\theta }\{\theta >\;0|(\frac{output}{\theta })\in {\mathrm{product}}^{\mathrm{meta}}(\mathrm{input})\}=\mathrm{MTE}(\mathrm{input},\;\mathrm{output})$$

The four clusters of mature, renewable, declining and growing technologies (*TE*^(*i*)^) are:

*TE^(i)^* = {(*input_i_*, *output_i_*)|*input_i_*, *output_i_* ≥ 0; *input_i_*→*output_i_*} (4)

The production probability (*i*) is:

*product^(i)^*(*input_i_*) = {*output_i_*|(*input_i_*, *output_i_*∈*TE^(i)^*} (5)

Group Technical Efficiency (*GTE*) equivalent to Group Leading Edge Distance Function (*DDF*^(*i*)^), as shown in Formula (6), where 0 ≤ *DDF*^(*i*)^(*input_i_*, *output_i_*) ≤ 1:

(6) $$\mathrm{DDF}{}^{\left(i\right)}({\mathrm{input}}_{i},\;{\mathrm{output}}_{i})=\inf_{\theta }\{\theta >\;0|(\frac{output_{i}}{\theta })\in \mathrm{producAppt}{}^{\left(i\right)}({\mathrm{input}}_{i})\}=\mathrm{GTE}({\mathrm{input}}_{i},\;{\mathrm{output}}_{i})$$

(4) Define the Technological Gap Ratio (TGR). *TGR* reflects the gap between common frontier and group frontier technology level. The higher the value, the closer the actual production efficiency is to the potential production efficiency [19], which means the higher the technology level [18]. When the input–output combination is (*input_i_*, *output_i_*), TGR can be shown in Formula (7), where 0 ≤ TGR (*input*_i_, *output_i_*) ≤ 1.

(7) $$TGR_{\left(input_{i},\;output_{i}\right)\;}=\frac{DDF^{meta}\left(input.output\right)}{DDF^{\left(i\right)}\left(input_{i},output_{i}\right)}\;=\frac{MTE\left(input,output\right)}{GTE\left(input_{i},output_{i}\right)}$$

Relationship between common frontier technology efficiency, group frontier technology efficiency and technology gap ratio can be expressed as:

MTE = GTE × TGR(8)

### 3.2. Variables Selection

#### 3.2.1. Input Variables

First, the labor factor. Labor input is an important part of urban economic and social development and plays a decisive role in improving the efficiency of resource allocation for the high-quality development of the mining city economy. Therefore, the total labor wage of mining cities is selected as the proxy variable of labor input.

The second is capital elements. Capital factors include land and plants, production equipment, mining-transportation-processing equipment, etc. Capital investment has a direct impact on the economic growth and resource allocation of mining cities. Usually, under certain conditions, the more capital factor resources are invested, the more productive activities there are, which is more conducive to improving the efficiency of resource factor allocation and promoting economic growth. Considering the availability of data, the gross fixed capital of mining cities is selected as the proxy variable of capital elements.

The third is resource elements. Resource input plays an important role in the development of mining cities, and resource input is the main way to obtain technical progress [13,15]. Based on the single method of processing the quantity of resource factors, this paper shows the input of resource factors by calculating the industrial concentration level of mining resource-based cities.

#### 3.2.2. Output Variables

The allocation of resource factors in the process of economic development of mining cities can not only bring economic output but also affect the input–output efficiency through the negative effect on the environment. The reason is that in the process of economic development of mining cities, the allocation capacity will be constantly adjusted, which can improve the technical level of resource and energy development and utilization and environmental governance capacity and play an important supporting role in resource reservation and environmental protection. It is an important way to solve the contradiction between population, resources and the environment and improve the carrying capacity of resources and the environment of mining cities to provide for sustainable growth and high-quality development of mining cities. Therefore, the output of resource allocation is further divided into economic output and environmental output.

First, economic output. The indicators of urban economic growth selected by the existing research are very rich, often involving multiple dimensions. By comparison, considering the representativeness and accessibility of indicators, the gross domestic product (GDP) of urban areas can more directly reflect the final output of the economic category.

The second is environmental outputs. Since the generation and discharge of pollutants have a large correlation with the economic level of the region, in terms of the selection of environmental indicators, the pollutant discharge indicators include production and living from the point of view of the pollution source, and there are gas pollution discharge, liquid pollution discharge and solid waste from the perspective of the material form. The environmental quality index is a composite value of environmental quality parameters and environmental quality standards. It also considers the emission and treatment of production and living pollution and is widely used to evaluate the treatment effect of environmental pollution. Therefore, considering the lack of data on solid waste, sulfur dioxide and wastewater discharge per capita are selected to measure environmental output.

#### 3.2.3. Data Sources and Descriptive Statistics of Variables

**Figure 1:**
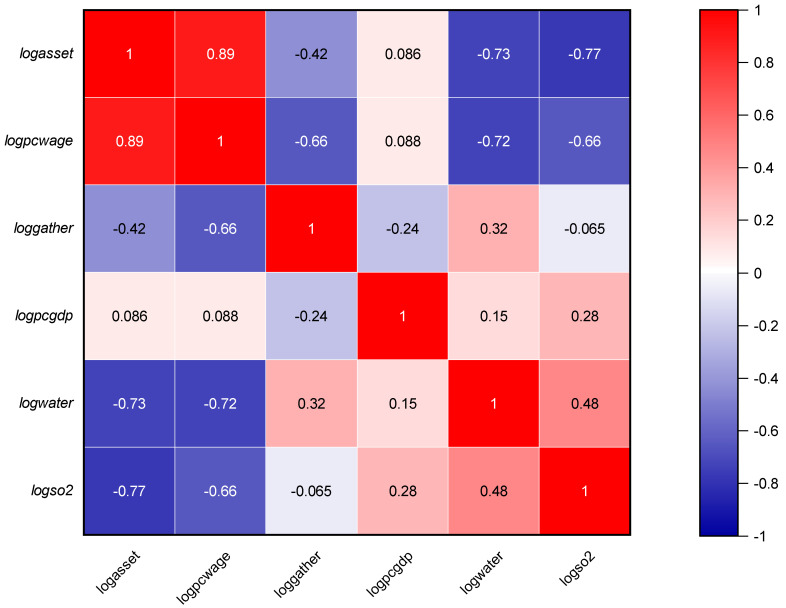
Correlation coefficient Note: Lower-triangular cells report Pearson’s correlation coefficients, upper-triangular cells are Spearman’s rank correlation.

In view of the availability of data, the sample data of mining-type cities used in this paper was selected from the list of resource-based cities (Schedule 1), and this study uses panel data from 99 mining cities in China (The list of resource-based cities is dynamically assessed and adjusted in combination with resource reserve conditions, development and utilization conditions, etc., and finally 99 representative cities are selected for analysis) (excluding Tibet, Hong Kong, Macao and Taiwan) from 2006 to 2019 for relevant statistics and quantitative analysis. Relevant data mainly come from China Urban Statistics Yearbook, China Macroeconomic Database, China Financial Statistics Database, China Urban Environment Database, etc. Detailed descriptive statistics and correlation analysis were conducted on the characteristics of relevant data samples, and the results are shown in Table 1 and Figure 1. It can be seen that the average values of human resources, financial resources, capital accumulation, industrial agglomeration, GDP and environmental output are 5.7569, 10.1789 and −0.4176 respectively after the logarithm is taken. Compared with the maximum and minimum values, the results show that the sample characteristics are close to the non-normal distribution. According to the standard deviation of sample data, the standard deviation of information resources and innovation output is 0.3473 and 0.1232 respectively, which indicates these variables data are characterized by relative concentration. The results show that there is no significant Multicollinearity between the main variables in Figure 1, which meets the operational requirements of the DEA model.

## 4. Analysis of Empirical Results

According to the constructed index system of high-quality input–output allocation efficiency of the mining city economy, the DEA-Meta-frontier model is comprehensively used to calculate the input–output allocation MTE, GTE and technology gap ratio TGR of the high-quality development of the mining resource-based city economy under the common frontier and the group frontier and to analyze the evolution characteristics of time-space differences. The descriptive statistics calculated by MaxDEA7.16 software are shown in Table 2.

### 4.1. Comprehensive Efficiency Analysis

#### 4.1.1. MTE and GTE

From the comparison of common frontier technology efficiency (MTE) and group frontier technology efficiency (GTE) in different regions (as shown in Table 2), MTE and GTE are distance function values of DMU based on the common and group boundaries, respectively, reflecting the distance from actual output to the common and group boundary output under the same input level [21].

First, the efficiency of high-quality input–output allocation of regional mining cities in China under the common frontier and group frontier is 0.5046 and 0.2806, respectively, indicating that the input factors of high-quality input–output allocation of China’s mining resource-based cities still have 49.54% saving space if the national optimal technology is used as a reference, and the saving space is as high as 71.94% if the regional optimal technology is taken as reference. The difference in the results is mainly due to the difference in the technical reference set between the two. The common frontier takes the potential optimal input–output allocation efficiency of all samples as the reference structure front, while the group frontier takes the mature, growth, regeneration and decline types as the grouping of the high-quality input–output allocation efficiency of the mining city economy.

Second, the MTE of growth is 0.6374, which indicates that there will be 36.26% efficiency improvement space for growing mining cities and 47.69%, 47.95% and 52.62% efficiency improvement space for growing mining cities. It can be seen that, in comparison, the high-quality input–output allocation efficiency of the regenerative, recession and mature mining cities is still low, which indicates that the elements of these three types of mining resource-based cities are relatively extensive, and the effective development and utilization are insufficient. At the same time, there may be a certain problem of "resource waste" or insufficient input of resource elements.

Third, the mean value of GTE from high to low was the recession type, growth type, regenerative type and mature type, with efficiency improvement spaces of 18.73%, 20.65%, 33.91% and 44.34%, respectively. The calculation results of GTE show that there is significant heterogeneity in the distance between the input–output efficiency of four different types of mining cities and their respective boundaries. Unlike the MTE calculation results, the input–output efficiency of the declining mining cities is relatively average, while the input–output efficiency of the mature mining cities with high-quality economic development varies greatly. The efficiency of the four types of mining city groups needs to be improved less than that of the common frontier efficiency, which indicates that from the perspective of groups, the input scale of resource elements within each group is more appropriate, but the balance of input and output allocation between groups is still needed.

Finally, compared with the two frontiers (MTE and GTE), the high-quality input–output allocation efficiency of the growing mining cities has little difference, while the two frontiers of the mature mining cities have the largest difference. In terms of the ranking of different frontier types, the ranking of the economic high-quality input–output allocation efficiency of growth and mature mining cities has not changed, but the ranking of the input–output allocation efficiency of declining and regenerative mining cities has undergone significant changes. The reason for this phenomenon may be that the resource input, innovation ability, macroeconomic level and educational development level of the growing and mature mining cities are superior to other types, which represents the national optimal level and the attraction ability is higher than other areas, and the technology collection under the two frontiers is basically the same, while the technology collection under the two frontiers of the declining and regenerative mining cities is significantly different, and the distance between the frontiers of the two types changes greatly.

#### 4.1.2. TGR

In terms of TGR, TGR reflects the gap between the technical level of the group and the technical level of the potential common boundary caused by the specific group institutional environment. The higher the TGR is, the closer the actual technical level of the DMU is to the common boundary technical level, that is, the higher the technical level under the corresponding institutional environment conditions. According to the mean value of TGR of population, mature type, regenerative type, declining type and growing mining cities in the sample period (as shown in Table 2), the mean value of TGR of four types of mining cities is less than 1, among which the mean value of TGR of mature mining cities in the sample period is 0.8432, close to 1, at a higher level, indicating that the technical level of mature mining cities is close to the common technical frontier, and reaches the potential common boundary technical level, which is 84.32% of the potential input–output allocation efficiency; However, the mean value of TGR of regenerative, declining and growing mining cities is below 0.8, which is relatively far away from the common technological frontier. Among them, the mean value of TGR of declining mining cities is 0.6267, indicating that it only reaches 62.67% of the potential input–output allocation efficiency, and there is still 37.33% of efficiency improvement space. At the same time, the differences in the TGR means of the four types of mining cities also explain the rationality of group division in this study.

As a whole, on the one hand, the efficiency of input–output allocation of high-quality economic development of the overall national mining cities and four types of mining cities under the common frontier is not as effective as DEA, and there is certain space for efficiency improvement; on the other hand, the efficiency of MTE and GTE of four types of mining cities is the best performance is growth, and the worst is mature type. This indicates that the growing mining cities have the strongest ability of high-quality input–output allocation and the highest degree of effective use of resource elements under the framework of sustainable development. Comparatively speaking, mature mining cities need to improve the ability of input–output allocation to avoid resource waste.

#### 4.1.3. Differences Types of Mining City

Third, from the comparison of the average value of high-quality input–output allocation efficiency of the four types of mining cities (as shown in Appendix A Table A1), the comprehensive efficiency of high-quality input–output allocation of mature mining cities is the best in Daqing and Dongying, the high-quality input–output allocation efficiency of Daqing and Dongying under the common frontier and group frontier is the first, and Daqing and Dongying are also the provinces and cities with the highest MTE average value among 99 mining resource-based cities, reaching 0.93461 and 0.9108, close to DEA efficiency. The worst performance in the common frontier was Yuncheng city and Baise city, with MTEs of 0.25953 and 0.29452, respectively, ranking last and second; that is, compared with the common boundary technology level of all samples, Yuncheng city and Baise city still had 74.047% and 70.548% efficiency improvement space. Yuncheng city and Baise city still had the worst performance in the group, with mean GTEs of 0.31539 and 0.34009, respectively. That is, mature mining cities have the highest input–output allocation efficiency of mining cities and have the lowest economic high-quality development input–output allocation efficiency. By analogy, the high-quality input–output allocation efficiency of regenerative, recession and growing mining cities can also be further analyzed.

#### 4.1.4. Time Change Trend

**Figure 2:**
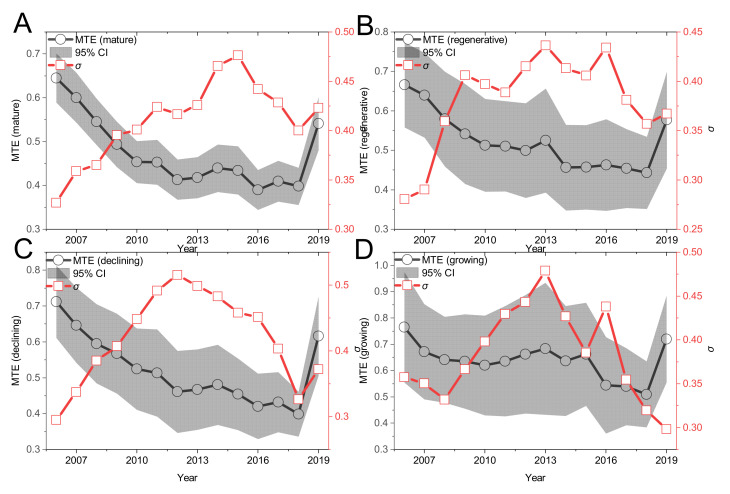
The trend of MTE in four types of mining cities from 2006 to 2019. Note: (**A**–**D**) represent mature, renewable, declining and growing mining cities respectively. The shaded area represents the 95% confidence interval (CI). σ Represents the convergence trend of MTE annual mean [22].

First, under the common front (as shown in Figure 2), the high-quality input–output allocation efficiency (MTE) of the four types of mining cities all shows the V-shaped change trend of decreasing first and then gradually increasing, but the decreasing range is distinct. Among them, the decline range of annual MTE change trend of mature mining cities is 2006–2016, with a slight recovery in the middle, and the bottom is 2016; the decline range of the annual MTE change trend of regenerative mining cities is 2006–2018, and the bottom is 2018; the annual MTE change trend of recession mining cities is consistent with that of regenerative mining cities, and the decline range coincides with that of regenerative mining cities; the decline range of the MTE annual average change trend of growing mining cities is 2006–2010 and 2013–2018, with a slight recovery in the middle, and the bottom is 2018. Basically, the average MTE of three types of mining cities, mature, regenerative and declining mining cities, began to show a certain degree of improvement at the end of the “Eleventh Five-Year Plan” period, but during the “12th Five-Year Plan” period, there was a general downward trend, while in the “13th Five-Year Plan” period, there was a significant degree of improvement. Unlike the above three types of mining city MTE changes, the growing mining city MTE in the “12th Five-Year Plan” presents a certain upward trend. The change trend of the annual mean value of MTE in the 12th Five-Year Plan may be related to the sustainable development of resource-based cities in 2013.

**Figure 3:**
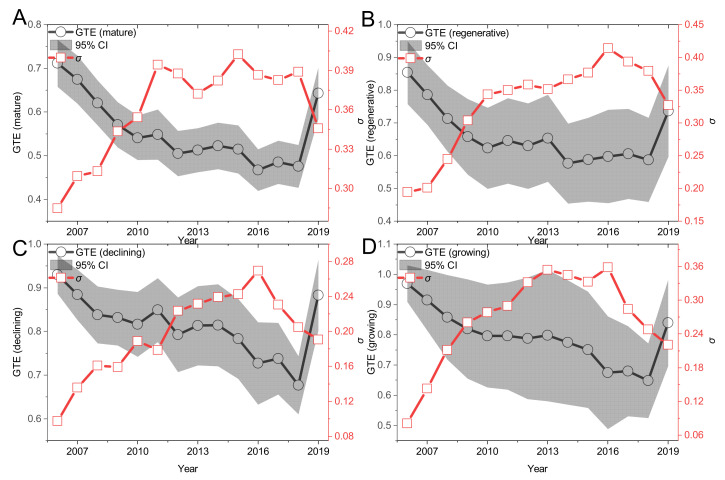
The Trend of GTE in Four Types of Mining Cities from 2006 to 2019. Note: (**A**–**D**) represent mature, renewable, declining and growing mining cities respectively. The shaded area represents the 95% confidence interval (CI). σ Represents the convergence trend of MTE annual mean [22].

Second, under the front of the group (as shown in Figure 3), the change law of GTE of the four types of mining cities shows similar change characteristics. Among them, the GTE of mature, renewable, declining and growing mining cities showed obvious fluctuation and decline trends before 2018; the GTE of mature and declining mining cities rebounded in 2011–2014; the GTE of renewable mining cities rose in 2014–2017; and the GTE of growing mining cities rebounded slightly in 2016–2017.

**Figure 4:**
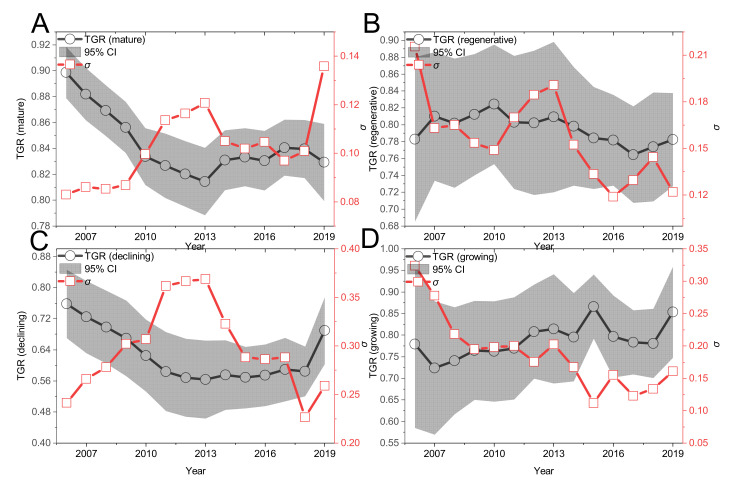
The trend of TGR of four types of mining cities from 2006 to 2019. Note: (**A**–**D**) represent mature, renewable, declining and growing mining cities respectively. The shaded area represents the 95% confidence interval (CI).σ Represents the convergence trend of MTE annual mean [22].

Finally, from the change trend of TGR of mature, regenerative, recession and growth type in the sample period (Figure 4), the TGR value of regenerative mining cities in 2006–2019 basically remains unchanged, with slight fluctuation in the middle and obvious “m” shape, indicating that the distance between the production front of input–output allocation and the common front in the high-quality economic development of regenerative mining cities basically remains unchanged. The mean value of the TGR of mature and declining mining cities mainly show a downward trend, and the change trend is consistent with the MTE change trend in Figure 2, indicating that the mature and declining mining cities are mainly caused by changes in MTE. The above two types of TGR indicate that the distance between the production frontier and the common frontier in the economic growth process of mining cities has been expanded to a certain extent, that is, the gap between the high-quality input–output allocation efficiency of the mining city economy and the potential high-quality input–output allocation efficiency of the mining city economy is expanding. The TGR value of the growing mining cities shows an obvious rising trend, which indicates that the distance between the production frontier and the common frontier in the process of high-quality economic development of the growing mining cities has a certain narrowing, that is, the gap between the high-quality input–output allocation efficiency of this type of mining cities and the potential high-quality input–output allocation efficiency of the mining cities is narrowing, and the high-quality input–output allocation efficiency of the growing mining cities has a catching-up effect on the high-quality economic growth of mature and regenerative mining cities.

### 4.2. Convergence Analysis

The convergence of economic development means that the difference in economic development shows a trend of gradually narrowing with time. To investigate the evolution trend of high-quality economic development differences of mining cities represented by MTE, GTE and TGR, the convergence model is used to test the convergence of MTE, GTE and TGR. Convergence is divided into σ and β convergence. The main principle of the σ convergence method is to judge the convergence by observing the distribution of the standard deviation of a variable between regions. The standard deviation decreases with time, which means that the difference in the variable between regions decreases, and there is σ convergence between regions. β convergence is developed from the convergence theory in the neoclassical growth model [23], and it is divided into absolute β convergence and conditional β convergence according to whether there are restrictions. The absolute β convergence assumes that each region has the same base of economic conditions, and a certain variable among regions will eventually reach the same steady growth rate and level. The conditional β convergence assumes that after considering the different economic basis conditions of each region, each region develops along its own steady growth path and finally reaches the steady growth rate and level. The main method of absolute β and conditional β convergence is to observe whether the efficiency change rate is related to the initial input–output allocation efficiency level by constructing a metrological equation. If there is a significant negative relationship, the regression coefficient β value is less than 0, indicating that the efficiency change rate is negatively related to the initial input–output allocation efficiency level. The higher the initial input–output allocation level is, the lower the efficiency change rate, or the lower the initial input–output allocation efficiency level is, the higher the rate of change. Mining cities with higher economic development input–output allocation efficiency levels have better development space. There is a faster growth rate than other mining cities at the early stage of development, and this type of β convergence exists in the high-quality economic development of mining cities.

#### 4.2.1. Space Inspection

Considering that the economic development of mining resource-based cities is not independent but has mutual influence, this paper tests the spatial agglomeration and spatial dependence degree [24] of common frontier efficiency, group frontier efficiency and the technical drop ratio of high-quality economic development of mining cities by measuring the overall Moran’s I index, and the specific calculation formula is as follows:

(9) $$\mathrm{Moran}’s\;I=\frac{n}{\sum_{i=1}^{n}\sum_{j=1}^{n}w_{ij}}\frac{\sum_{j=1}^{n}w_{ij}\left(x_{i}-\bar{x}\right)\left(x_{j}-\bar{x}\right)}{\sum_{i=1}^{n}{\left(x_{i}-\bar{x}\right)}^{2}}$$

where n is the total number of mining cities, $w_{ij}$ is the spatial weight matrix (using the spatial adjacency weight matrix, the adjacency is 1, and the nonadjacency is 0), and $x_{i}$ and $\bar{x}$ are the input–output allocation efficiency (MTE, GTE and TGR) and their mean values. According to the formula, the value range of Moran’s I index is [−1,1]. If Moran′s I is greater than 0, it indicates a positive correlation (adjacent mining cities with high economic quality development input–output allocation efficiency or adjacent mining cities with low economic quality development input–output allocation efficiency). A larger value indicates that the spatial correlation of economic development allocation efficiency of mining cities is stronger. If it is less than 0, it indicates a negative correlation (adjacent mining cities with high economic quality development input–output allocation efficiency and adjacent mining cities with low economic quality development input–output allocation efficiency). A smaller value indicates that the difference between the economic development allocation efficiency of mining cities is greater. If Moran’s I index tends to 0, it indicates that the economic development of mining cities has no spatial correlation.

**Figure 5:**
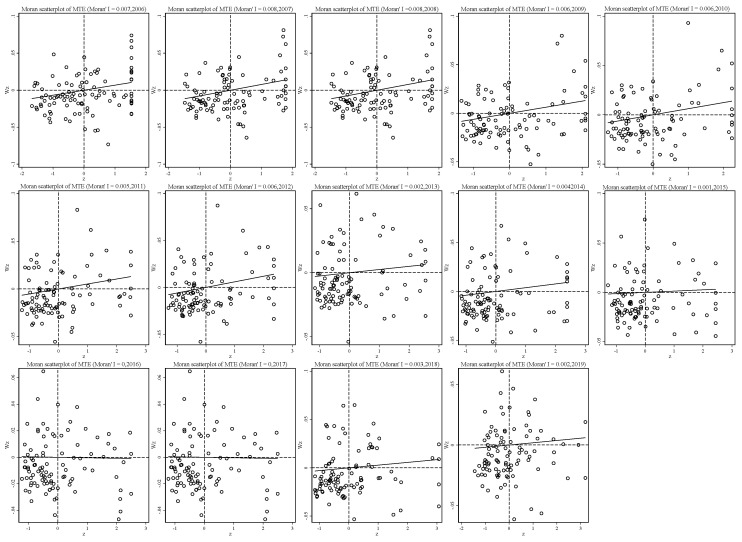
Distribution change of MTE Moran’s I scatter plot from 2006 to 2019.

**Figure 6:**
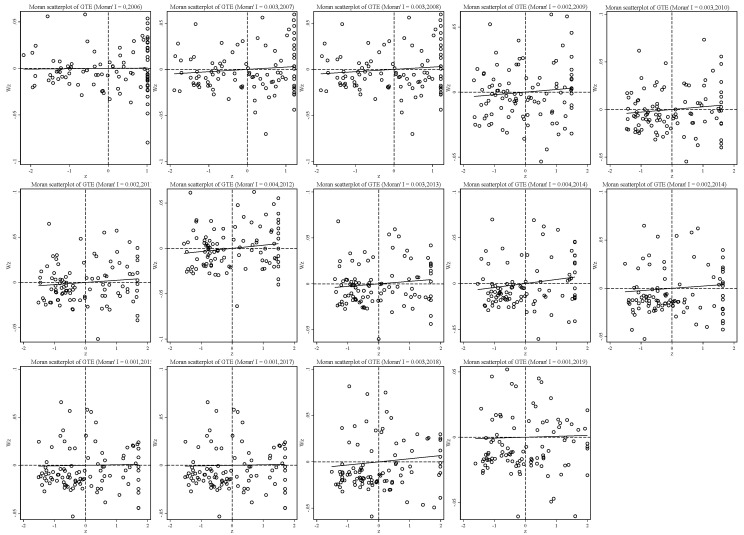
Distribution Change of GTE Moran’s I Scatter plot from 2006 to 2019.

**Figure 7:**
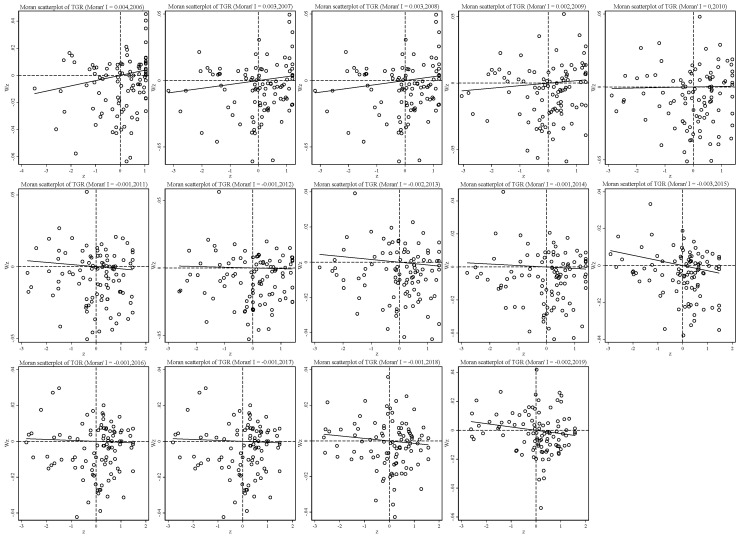
Distribution Change of TGR Moran’s I Scatter plot from 2006 to 2019.

As a result, the spatial Moran’s I indices of MTE, GTE and TGR are calculated, and the results are shown in Figure 5, Figure 6 and Figure 7. Figure 5 shows the global spatial autocorrelation of MTE in mining cities from 2006 to 2019 (Moran’s I). The results show that Moran’s I index is positive in the sample period, the fluctuation range is 0.0001~0.008, and all have passed the significance test, indicating that the common frontier efficiency of economic development among mining cities has a positive correlation during the sample period, demonstrating the phenomenon of spatial concentration on the whole; that is, the common technological frontier of high-quality economic development of mining cities is affected by the adjacent mining cities, the mining cities with high MTE are neighbors, and the cities with low MTE are neighbors. According to the temporal trend of Moran′s I index, the Moran’s I index of MTE showed a downward trend as a whole, but there was a slight fluctuation in the middle.

Figure 6 shows the global spatial autocorrelation value (Moran’s I) of the GTE of mining cities in 2006–2019. The results show that Moran’s I index is positive during the sample period, the variation trend is consistent with MTE, and the fluctuation range is 0.00001~0.004. All of them pass the significance test, which demonstrates that the group frontier efficiency of economic development among mining cities has a positive correlation in the sample period and shows the phenomenon of spatial agglomeration on the whole; that is, the technical frontier of the high-quality economic development group of mining cities is affected by the adjacent mining cities. Moreover, the mining cities with higher GTEs are neighbors to each other, and the cities with lower GTEs are neighbors to each other. According to the temporal trend of the Moran’s I index, the whole Moran’s I index of GTE shows a downward trend, but there is a slight fluctuation in the middle.

Figure 7 shows the global spatial autocorrelation value (Moran’s I) of the TGR of mining cities in 2006–2019. The results show that Moran’s I index fluctuates in the range of −0.003~0.004 during the sample period, and all of them pass the significance test, showing a significant downward trend. The Moran′s I index of TGR decreased from 0.004 in 2006 to 0.0001 in 2010, indicating that the technological gap of economic development between mining cities decreased compared with the phenomenon of spatial agglomeration in 2006–2010; the Moran′s I index of TGR in 2011–2019 was negative, that is, the technological gap of high-quality economic development of mining cities (catch-up trend) was affected by the neighboring mining cities, and mining cities with higher TGR and mining cities with lower TGR are neighbors to each other.

#### 4.2.2. σ. Convergence Analysis

**Figure 8:**
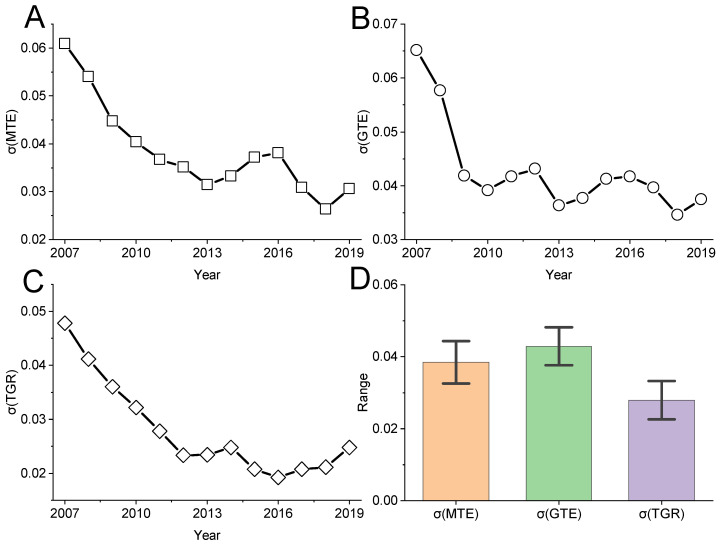
Annual trend of spatial σ convergence and descriptive statistical results. (**A**–**C**) represent σ convergence of MTE, GTE, TGR respectively. (**D**) represents the mean of σ convergence and its 95% confidence interval.

Figure 2, Figure 3 and Figure 4 all discuss the change trend of general σ convergence, and the results do not reach a clear conclusion that there is general σ convergence in the input–output allocation efficiency of high-quality development of the mining city economy. Considering the spatial autocorrelation among MTE, GTE and TGR, the spatial σ convergence characteristics of three kinds of efficiency are discussed in detail. Unlike the general σ convergence model calculation formula and process [22], spatial σ convergence needs to be transferred into the spatial weight matrix (the spatial weight edge is used to calculate the spatial weight of Moran’s I index). By deleting the missing value, the maximum likelihood estimation of the difference term and the lag of the variable is carried out. Finally, the standard deviation of the residual error of the regression result is calculated according to the time, and the change trend shown in Figure 8 is drawn. Figure 8 shows the temporal evolution trend of MTE, GTE and TGR standard deviations of input–output for high-quality economic development of mining cities to describe the spatial σ convergence of the mean value of input–output allocation efficiency for high-quality economic development of mining cities. As a whole, the standard deviation of the σ convergence of MTE, GTE and TGR in the sample period from 2006 to 2019 shows a downward trend. That is, there is significant spatial σ convergence in the growth of the input–output allocation efficiency of the high-quality economic development of mining cities, which cannot offset the internal downward trend, although some years have recovered. Specifically, the standard deviation of σ convergence in MTE, GTE and TGR space is relatively concentrated between [0.02,0.07], and the internal gap is in a slow narrowing trend: using 2013 as the watershed, the standard deviation of σ convergence in MTE, GTE and TGR space tends to decrease rapidly before 2013, and the internal gap is narrowed; after 2013, the standard deviation of σ convergence in MTE, GTE and TGR space rises slowly and then falls, and the internal gap is still in a narrowing trend. Figure 8D also shows that in 2006–2019, the highest standard deviation of the spatial σ convergence of mining resource-based cities is GTE, and the lowest is TGR, which shows that the gap between the technical drop ratio and mining cities is smaller, and the gap in GTE growth is gradually expanding. 

Since MTE, GTE and TGR have spatial σ convergence in 2006–2019, this paper will conduct a β test on MTE, GTE and TGR to determine the convergence of input–output allocation efficiency in the process of high-quality economic development of mining cities.

#### 4.2.3. β. Convergence Analysis

Assuming that the macroeconomic development environment, regional policy, financial environment and industrial structure faced by mining cities are consistent, the economic development level of the four different types of mining cities gradually converges to the same development level over time in the sample period, which is called absolute β convergence. Then, the general expression of the absolute β convergence test can be obtained (10):

(10) $$\frac{1}{T}\ln(\frac{x_{i,t}}{x_{i,t-1}})=\alpha +\beta \ln(x_{i,t-1})+\varepsilon$$

where $x_{i,t}$, $x_{i,t-1}$ represent the input–output allocation efficiency of high-quality economic development of mining cities in *t* and *t* − 1, respectively; α represents the constant term; β represents the convergence coefficient; and ε represents the error term. The formula for measuring the convergence of β is shown in (11):

(11) $$\beta =-\frac{1-e^{-\lambda T}}{T}$$

where *λ* is the rate of convergence. If β < 0, it means that there is convergence in the input–output allocation efficiency of the high-quality economic development of the mining city, that is, the development of the input–output allocation efficiency of the high-quality economic development of the mining city is converging; if β > 0, it means that the efficiency of input–output allocation for high-quality economic development of mining cities does not have convergence. Because the spatial test results show that the input–output allocation efficiency of the high-quality economic development of mining cities has global spatial autocorrelation, due to the economic development of each mining city is not isolated but will be affected by the economic development of external regions, it is necessary to introduce the concept of spatial correlation to analyze the convergence of the input–output allocation efficiency of the high-quality economic development of mining cities. According to Formula (10), this paper constructs a spatial panel regression model with spatial absolute β convergence. The specific expression is as follows:

(12) $$\frac{1}{T}\ln\left(\frac{x_{i,t}}{x_{i,t-1}}\right)=\alpha +\beta \ln\left(x_{i,t-1}\right)+\beta w_{i,j}\ln(\frac{x_{i,t}}{x_{i,t-1}})+\varepsilon$$

where, $\ln(\frac{x_{i,t}}{x_{i,t-1}}$) represents the allocation efficiency growth rate of mining city *i* in period *t,* *ρ* is the spatial regression coefficient, and *W_i,j_* is the spatial weight matrix. Therefore, it can reflect the efficiency of input–output allocation for the high-quality development of the mining city economy. The expression for the convergence rate is:

(13) $$\gamma \;=-\frac{1}{T}\ln\left(1+\beta \right)$$

For the accuracy of the model, the parameter estimation method adopted in this paper is the maximum likelihood method. When the spatial error model (SEM) or the spatial lag model (SLM) is selected, the test method is the LM test. SLM or SEM is determined by comparing IMlag and LMerr statistics. Because the LMlag statistics calculated by each mining city in this paper are higher than LMerr statistics, the results of the Hausmann test also point to the use of fixed effects. Therefore, the fixed-effect spatial lag model (SLM) is used to investigate the convergence characteristics of the input–output allocation efficiency of high-quality economic development in the mining cities. Table 3 shows the absolute β convergence results of the OLS model and the spatial lag model for the high-quality development input–output allocation efficiency of the mining city economy in 2006–2019.

Table 3 shows that the regression coefficient β of the input–output allocation efficiency of the high-quality development of the mining city economy is negative and significant at the 1% level when all research samples are tested for convergence, which indicates that the input–output allocation efficiency of the high-quality development of the mining city economy converges significantly during the sample period, there is an absolute convergence trend on the whole, and the growth gap of the input–output allocation efficiency of the high-quality development of the mining city economy gradually narrows. In terms of classification, the β values of the input–output allocation efficiency of the high-quality development of mature, regenerative, declining and growing mining cities are significantly negative and are all significant at the 1% level, indicating that the internal gap in the input–output allocation efficiency of the high-quality development of the four types of mining cities has a narrowing trend. The conclusion of the absolute β convergence test shows that the gap in the input–output allocation efficiency of high-quality economic development in mining cities is gradually narrowing. From a more accurate point of view, we can think that the efficiency of input–output allocation of high-quality economic development of mining cities presents the characteristics of “convergence”, and the growth gap of input–output allocation efficiency of four types of high-quality economic development of mining cities is gradually narrowing.

From the national level, the difference in the input–output allocation efficiency of the high-quality development of the mining city economy generally narrows, but the convergence speed is very slow, 0.0308, 0.0343 and 0.0271. From the perspective of the four types of mining cities, the common frontier efficiency of input–output allocation of high-quality development of mature, regenerative, declining and growing mining city economy (MTE) passes the absolute β convergence test, and the convergence speed is significantly different, 0.0481, 0.0281, 0.0324 and 0.0294, respectively. Consistent with the convergence change of MTE, the convergence speed of GTE is 0.0477, 0.0299, 0.0448, 0.0345, and the convergence speed of TGR is not much different. The convergence speed of the TGR of the high-quality input–output configuration of the four types of mining cities is 0.0330, 0.0326, 0.0205, and 0.0277. Therefore, the high-quality input–output allocation efficiency of the overall and four types of mining cities will eventually converge to the same value.

### 4.3. Decomposition of High-Quality Input-Output Allocation Inefficiency of Mining Cities in Different Regions

Although the Meta-Frontier method is used to estimate the common frontier efficiency MTE, group efficiency GTE and technical gap ratio TGR of high-quality input–output allocation of regional mining cities, it is impossible to determine the efficiency difference and the true root of inefficiency among the mining cities and by the mining city type. However, the numerical difference in TGR provides a solution for distinguishing the root causes of the high-quality input–output allocation inefficiency of different mining cities [25,26]. Some scholars have found that the main reasons for the current efficiency loss are the gap in cutting-edge technology and the management inefficiency caused by group management decisions. Based on the research ideas of [10], the inefficiency of high-quality input and output allocation of 99 mining cities is decomposed into the inefficiency of the production technology gap caused by the group and common frontier TGRI (Technology Gap Ratio Inefficiency, TGRI) and the inefficiency of management within the group Group-specific Managerial Inefficiency (GMI). TGRI and GMI together constitute the total inefficiency loss meta-frontier total inefficiency (MTI), as shown in Formulas (14)–(16), and the results are shown in Appendix A Table A2.

(14) $$TGRI_{n}^{i}=GTE_{n}^{i}\times \left(1-TGR_{n}^{i}\right)$$

(15) $$GMI_{n}^{i}=1-GTE_{n}^{i}$$

(16) $$MTI_{n}^{i}=TGRI_{n}^{i}+GMI_{n}^{i}$$

It needs to be explained that, in combination with the actual characteristics of high-quality input–output allocation of the mining city economy and referring to the research idea of [26], when the proportion of TGRI is obviously higher (more than 70%), it needs to improve the efficiency of regional investment allocation by improving the group environment, while when the proportion of GMI is higher (more than 70%), it needs to improve the efficiency level by improving the high-quality input–output allocation capacity of the regional mining city economy. When the proportion of TGRI and GMI is equal, it indicates that the group needs to improve the innovation environment and improve the high-quality input–output allocation capacity of regional mining cities [27].

First, from the point of view of type, the loss of invalidity rate and improvement path of different groups have obvious heterogeneity. From 2006 to 2019, the contribution degree of mature mining cities was 12.8400% for TGRI, 44.3448% for GMI and 57.1847% for MTI, which indicates that the key to improving the efficiency of input–output allocation is to further improve the allocation capacity of mining cities. The average contribution degrees of the TGRI, GMI and MTI of regenerative mining cities are 13.2444%, 33.9072% and 47.1516%, respectively, which also indicates that the optimization of input and output factors is the most important for the high-quality development of regenerative mining cities. The average contribution degrees of the TGRI, GMI and MTI of declining mining cities are 10.9328%, 18.7332% and 29.6660%, respectively. The results show that the contribution degrees of TGRI and GMI have little difference, which is very important for the high-quality economic development of declining mining cities, the overall improvement of resource factor input–output allocation capacity and the improvement of the group environment. The results of the growing mining cities are similar to those of the regenerative and mature ones, but the contribution of TGRI and GMI of the growing mining cities is not distinct, that is, in the process of high-quality economic development of the growing mining cities, it is mainly to optimize the input–output allocation capacity of resource elements, but it is necessary to improve the group environment.

Second, from the perspective of urban distribution, there is obvious heterogeneity in the loss of urban inefficiency and the improvement path. The contribution degree of TGRI and GMI is lower than 70%, but the ineffective loss degree of some mining cities is close to 70%. From the mean, the contribution of TGRI is much lower than that of GMI. The cities whose GMI contribution is close to 70% are Yuncheng, Baise, Zhangjiakou, Xingtai, Weinan, Hechi and Changzhi. These mining cities are mature, and the key to improving the efficiency of high-quality input–output allocation is the ability of input–output allocation. The cities with similar contribution ratios of GMI and TGRI include Baoshan, Chizhou, Daqing, Datong, Handan, Jixi, Jincheng, Loudi and Yichun, Ordos, Nanchong, Shuozhou and Yulin, Hegang, Puyang and Wuhai, Baotou, Huludao, Lijiang, Tonghua and Xuzhou. These TGRI and GMI are basically the same degree of inefficiency, belonging to provinces and cities that need to improve the regional innovation environment and improve the high-quality input–output allocation capacity of regional mining cities. 

## 5. Conclusions 

With the in-depth implementation of the National Sustainable Development Plan for Resource-based Cities (2013–2020), the scale, structure and intensity of high-quality input–output allocation of China’s mining cities have been improved to a certain extent. However, there are also some phenomena and problems in which the efficiency of resource allocation is not high, which restricts the sustainability of high-quality economic development. In this paper, the DEA meta-frontier method is used to estimate the allocation efficiency of high-quality input and output under the common frontier and group frontier of 99 mining cities in China from 2006 to 2019, and the convergence of the allocation efficiency and the future improvement path are discussed. The results show the following (This conclusion is accurate in China, but it may be the opposite in other countries. We will discuss the high-quality development of mining cities in a larger scale for further research).

First, in terms of the efficiency and technology gap ratio of the common frontier and group frontier, (1) the input factors of the high-quality input–output allocation of national regional mining cities under the common frontier and group frontier has 49.54% and 71.94% saving space, respectively. (2) Under the common frontier, the average value of MTE from high to low is growth, regenerative, declining, and mature. In comparison, regenerative, declining, and mature mining cities still have low economic high-quality input–output allocation efficiency. There may be a certain “resource waste” problem or there may be insufficient input of resource elements.

Second, the common frontier efficiency of economic development among mining cities has a positive correlation in the sample period, and it shows the phenomenon of spatial agglomeration on the whole. The standard deviation of the spatial σ convergence of MTE, GTE and TGR in the sample period from 2006 to 2019 shows a downward trend; that is, there is a significant spatial σ convergence in the increase in the input–output allocation efficiency of high-quality economic development of mining cities. Although some years have a rise, it cannot offset the internal downward trend. From the national level, the difference in the input–output allocation efficiency of high-quality economic development in mining cities is narrowing on the whole, but the convergence speed is very slow; the convergence speed of the input–output allocation efficiency of high-quality economic development in mature, regenerative, declining and growing mining cities is quite different.

Third, in view of the high quality of mature mining city economy, the key to improving the efficiency of input–output allocation is to further improve the allocation capacity of the mining city; optimizing the allocation of the input–output factors is the most important to achieve high-quality development of regenerative type; for recession type, it is very important to comprehensively improve the allocation capacity of input–output of resource factors and improve the group environment; in the process of high-quality development of growing mining city economy, it is mainly to optimize the allocation capacity of input–output of resource factors, but it is necessary to improve the group environment.

Combined with the characteristics of high-quality input–output allocation of mining cities, the policy implications include the following:

One is to comprehensively strengthen cross-regional sustainable cooperation among mature, renewable, declining and growing mining cities. We should give full play to the leading radiation and source supply role of the national mining city regeneration demonstration zone, explore the establishment of a long-term mechanism to improve win–win cooperation, and improve the level and level of cross-mining city type cooperation. Efforts should be made to promote the construction of a cross-regional system of recession-type and regeneration-type key mining cities and growing mining cities and to explore the benefit-sharing mechanism and win–win cooperation mode of the orderly transfer of resource elements along four types of plate gradients. 

The second is to build a mining city policy consortium around the common scientific issues of sustainable development. On the one hand, we will adhere to the combination of central planning and local responsibilities, fully mobilize local authorities to optimize the high-quality economic development environment of mining cities and improve the allocation capacity of input and output factors; on the other hand, we will promote the establishment of joint research centers and other resource condition platforms among mining cities, carry out joint tackling of key technologies common to the sustainability of mining cities, and encourage and support the formulation of joint sustainability policies and measures among regions.

The third is to coordinate the high-quality input–output allocation of mature, renewable, declining and growing mining cities. On the one hand, it is necessary to improve the development and utilization efficiency of resource elements of different types of mining cities and accelerate the conversion of new and old kinetic energy. On the other hand, at the top-level design level, the allocation of high-quality input and output of the mining city economy needs to be prioritized to declining cities, adhere to the combination of “blood supply” and “hematopoiesis”, and continuously improve the resource agglomeration function and ecological environment of declining mining cities to improve the economic high-quality input–output allocation capacity of declining mining cities.

Finally, it is necessary to optimize the mutual aid mechanism of different types of mining cities and explore and establish a perfect support mechanism of mature mining cities to grow and regenerate mining cities in order to promote the reasonable flow of talent, technology and funds to grow declining mining cities.

## Figures and Tables

**Table 1:** The summary statistics.

Var	Name	Obs	Mean	SD	Min	Median
logasset	Fixed assets (based 2000)	1386	5.7569	1.064	2.782	5.852
logpcwage	Per capita salary	1386	10.1789	0.589	2.283	10.25
loggather	Industrial agglomeration	1386	−0.4176	0.585	−3.391	−0.3417
logpcgdp	Per GDP	1386	9.3490	0.593	3.943	9.298
logwater	Per wastewater discharge	1386	2.5903	0.816	−0.07886	2.59
logso2	Per SO_2_ emissions	1386	−4.1559	1.024	−7.627	−4.072

**Table 2:** Statistical description of regional innovation resource allocation efficiency from 2006 to 2019.

City Type	MTE					GTE					TGR				
	Mean	Min	S.D.	Max	Range	Mean	S.D.	Min	Max.	Range	Mean	S.D.	Min	Max	Range
Mature type	0.4738	0.1740	0.4126	1	0.8260	0.5566	0.2053	0.5019	1	0.7947	0.8432	0.4917	0.8418	1	0.5083
Regenerative	0.5231	0.2191	0.4757	1	0.7809	0.6609	0.2276	0.6236	1	0.7724	0.7951	0.4396	0.7866	1	0.5605
Decay type	0.5205	0.1680	0.4602	1	0.8320	0.8127	0.3757	0.8328	1	0.6243	0.6267	0.2919	0.5778	1	0.7081
Growth	0.6374	0.2240	0.6747	1	0.7760	0.7935	0.2668	0.8890	1	0.7332	0.7885	0.3242	0.7990	1	0.6758
National	0.5046	0.1680	0.4434	1	0.2806	0.6420	0.2053	0.6070	1	0.4082	0.7899	0.2919	0.8146	1	0.1672

Note: 1. S.D. is Standard deviation. 2. Summarize and sort according to the calculation results of MaxDEA7.16 software.

**Table 3:** Summary of absolute β convergence results of input-output allocation efficiency space for high-quality economic development of mining cities.

Vars	(1) OLS	(2) FE	(3) RE	(4) FE	(5) FE	(6) FE	(7) FE
	Overall	Overall	Overall	Growth	Mature	Decay	Regenerative
Panel A							
l.MTE	−0.129 ***	−0.350 ***	−0.129 ***	−0.490 ***	−0.325 ***	−0.365 ***	−0.337 ***
	(0.0128)	(0.0212)	(0.0128)	(0.0783)	(0.0267)	(0.0500)	(0.0605)
Constant	0.0570 ***	0.167 ***	0.0570 ***	0.306 ***	0.144 ***	0.180 ***	0.168 ***
	(0.00699)	(0.0109)	(0.00699)	(0.0504)	(0.0130)	(0.0265)	(0.0320)
λ	0.0099	0.0308	0.0099	0.0481	0.0281	0.0324	0.0294
Obs	1287	1287	1287	117	741	247	182
R2	0.073	0.186		0.268	0.178	0.190	0.156
Panel B							
l.GTE	−0.122 ***	−0.381 ***	−0.122 ***	−0.487 ***	−0.342 ***	−0.466 ***	−0.383 ***
	(0.0131)	(0.0218)	(0.0131)	(0.0699)	(0.0287)	(0.0555)	(0.0527)
Constant	0.0715 ***	0.236 ***	0.0715 ***	0.375 ***	0.183 ***	0.373 ***	0.242 ***
	(0.00887)	(0.0142)	(0.00887)	(0.0559)	(0.0162)	(0.0453)	(0.0354)
λ	0.0093	0.0343	0.0093	0.0477	0.0299	0.0448	0.0345
Obs	1287	1287	1287	117	741	247	182
R2	0.063	0.204		0.312	0.172	0.237	0.241
Panel C							
l.TGR	−0.108 ***	−0.316 ***	−0.108 ***	−0.370 ***	−0.366 ***	−0.249 ***	−0.321 ***
	(0.0119)	(0.0210)	(0.0119)	(0.0728)	(0.0296)	(0.0464)	(0.0504)
Constant	0.0814 ***	0.246 ***	0.0814 ***	0.296 ***	0.303 ***	0.150 ***	0.255 ***
	(0.00956)	(0.0167)	(0.00956)	(0.0575)	(0.0251)	(0.0293)	(0.0404)
λ	0.0082	0.0271	0.0082	0.0330	0.0326	0.0205	0.0277
Obs	1287	1287	1287	117	741	247	182
R2	0.060	0.160		0.195	0.182	0.112	0.195

Note: Values in brackets are standard error values, Panel A represents sample data of MTE, Panel B represents sample data of GTE and Panel C represents sample data of TGR. *** indicate statistical significance at 1%.

## Data Availability

Publicly available datasets were analyzed in this study. These datasets can be found here: https://data.stats.gov.cn/ (accessed on 18 March 2022).

## Appendix A

**Table A1:** Statistical description of input-output allocation efficiency of mining cities from 2006 to 2019.

Sort	City	MTE	Rank	GTE	Rank	TGR	Rank	Sort	City	MTE	Rank	GTE	Rank	TGR	Rank
Mature	Anshun	0.5275	32	0.5488	63	0.7239	76	Mature	Yichun	0.3993	65	0.4507	80	0.4938	96
Mature	Baise	0.2945	97	0.3401	98	0.8031	60	Mature	Yunfu	0.3472	84	0.4208	90	0.8506	35
Mature	Baoji	0.3509	83	0.4155	91	0.6997	80	Mature	Yuncheng	0.2595	99	0.3154	99	0.7176	78
Mature	Baoshan	0.4395	54	0.5367	67	0.6034	88	Mature	Zhangjiakou	0.2953	96	0.3656	97	0.8142	51
Mature	Benxi	0.4911	41	0.5585	60	0.7987	61	Mature	Changzhi	0.3140	93	0.3962	93	0.8312	40
Mature	Bozhou	0.6501	21	0.7941	29	0.7778	68	Mature	Chongqing	0.3343	90	0.4112	92	0.6899	83
Mature	Chenzhou	0.4487	52	0.5512	61	0.7202	77	Mature	Zigong	0.5809	25	0.6294	45	0.8318	39
Mature	Chengde	0.3970	68	0.5383	66	0.8147	50	Growth	Erdos	0.8338	9	0.9380	7	0.8096	55
Mature	Chizhou	0.4319	57	0.4904	73	0.4360	99	Growth	Hezhou	0.4788	44	0.6455	41	0.7924	66
Mature	Chifeng	0.4718	46	0.5967	53	0.8262	43	Growth	Hulunbeier	0.6646	19	0.8336	24	0.8240	45
Mature	Chuzhou	0.5067	36	0.5845	55	0.7967	64	Growth	Liupanshui	0.2970	95	0.4255	86	0.8107	53
Mature	Dazhou	0.4795	43	0.5817	56	0.8105	54	Growth	Nanchong	0.8793	6	0.9179	13	0.9790	2
Mature	Daqing	0.9346	2	0.9538	4	0.7981	63	Growth	Shuozhou	0.7055	15	0.9739	2	0.8710	22
Mature	Datong	0.3650	77	0.4291	85	0.4506	98	Growth	Songyuan	0.8204	11	0.9378	8	0.7505	73
Mature	Dongying	0.9108	4	0.9202	12	0.8346	37	Growth	Xianyang	0.3384	88	0.5968	52	0.8253	44
Mature	Ezhou	0.5724	26	0.6319	44	0.7363	74	Growth	Yulin	0.7193	14	0.8723	18	0.9616	6
Mature	Ganzhou	0.4150	62	0.4613	77	0.5921	89	Decay	Baishan	0.5235	33	0.8642	21	0.8619	29
Mature	Guang’an	0.8861	5	0.9167	14	0.8600	30	Decay	Fushun	0.4578	50	0.8732	17	0.8923	14
Mature	Guangyuan	0.6178	23	0.6583	39	0.7948	65	Decay	Fuxin	0.4756	45	0.7297	33	0.8400	36
Mature	Handan	0.3827	71	0.4947	72	0.4836	97	Decay	Hegang	0.8140	12	0.9093	16	0.8924	13
Mature	Hechi	0.3360	89	0.3956	94	0.9685	4	Decay	Huaibei	0.4257	59	0.6241	47	0.7987	62
Mature	Hebi	0.3852	70	0.4567	78	0.5908	90	Decay	Huang	0.3443	85	0.6998	36	0.8176	48
Mature	Heihe	0.8431	8	0.8694	19	0.9346	9	Decay	Jiaozuo	0.3721	74	0.8423	23	0.9638	5
Mature	Hengyang	0.3650	76	0.4414	82	0.9709	3	Decay	Jingdezhen	0.3398	87	0.6645	38	0.8043	59
Mature	Huzhou	0.3928	69	0.4530	79	0.8916	15	Decay	Liaoyuan	0.6839	16	0.9666	3	0.8327	38
Mature	Huainan	0.3815	72	0.4435	81	0.6896	84	Decay	Luzhou	0.3776	73	0.7363	31	0.8068	57
Mature	Jixi	0.7507	13	0.8107	27	0.9226	10	Decay	Pingxiang	0.4140	63	0.7009	35	0.8809	18
Mature	Jilin	0.4166	61	0.5129	71	0.6891	85	Decay	Puyang	0.5388	30	0.9163	15	0.9901	1
Mature	Jining	0.3702	75	0.4775	75	0.7037	79	Decay	Qitaihe	0.8247	10	0.9207	11	0.8699	24
Mature	Jincheng	0.4478	53	0.5248	68	0.5176	94	Decay	Shaoguan	0.2751	98	0.5494	62	0.9552	7
Mature	Laiwu	0.5073	35	0.6356	43	0.6438	87	Decay	Tongling	0.6576	20	0.9323	10	0.7921	67
Mature	Lincang	0.4248	60	0.5620	59	0.7346	75	Decay	Wuhai	0.9535	1	0.9788	1	0.9529	8
Mature	Linfen	0.3546	81	0.4252	87	0.6929	82	Decay	Xinyu	0.4361	55	0.8501	22	0.8235	46
Mature	Longyan	0.4977	39	0.5696	57	0.9156	11	Decay	Yichun	0.6104	24	0.8666	20	0.7760	69
Mature	Loudi	0.3972	67	0.4391	83	0.5219	93	Decay	Zaozhuang	0.3647	78	0.8157	26	0.8648	28
Mature	Mudanjiang	0.4983	38	0.6118	49	0.8716	20	Regenerative	Anshan	0.5059	37	0.6218	48	0.8695	25
Mature	Nanping	0.4534	51	0.5232	69	0.6605	86	Regenerative	Baotou	0.6396	22	0.9341	9	0.8710	21
Mature	Panzhihua	0.5220	34	0.6534	40	0.8115	52	Regenerative	Huludao	0.3516	82	0.4830	74	0.5074	95
Mature	Pingdingshan	0.3342	91	0.4219	89	0.8966	12	Regenerative	Lijiang	0.8443	7	0.9408	6	0.8048	58
Mature	Qujing	0.3550	80	0.4361	84	0.6946	81	Regenerative	Linyi	0.4604	49	0.5468	64	0.8699	23
Mature	Sanmenxia	0.4121	64	0.5206	70	0.8880	17	Regenerative	Luoyang	0.4357	56	0.6259	46	0.8551	34
Mature	Sanming	0.3579	79	0.4251	88	0.7694	71	Regenerative	Ma’anshan	0.3989	66	0.5950	54	0.8095	56
Mature	Shaoyang	0.5286	31	0.8210	25	0.8655	27	Regenerative	Nanyang	0.4948	40	0.5996	51	0.8691	26
Mature	Suzhou	0.5499	29	0.7324	32	0.7579	72	Regenerative	Panjin	0.9199	3	0.9461	5	0.8756	19
Mature	Taian	0.5652	28	0.6822	37	0.8563	33	Regenerative	Suqian	0.5714	27	0.7100	34	0.8571	31
Mature	Weinan	0.3232	92	0.3753	95	0.8148	49	Regenerative	Tangshan	0.4636	48	0.6040	50	0.8292	41
Mature	Xingtai	0.3033	94	0.3748	96	0.7717	70	Regenerative	Tonghua	0.3440	86	0.4622	76	0.5223	92
Mature	Xuancheng	0.4855	42	0.5627	58	0.8903	16	Regenerative	Xuzhou	0.4275	58	0.5440	65	0.5890	91
Mature	Ya’an	0.6728	17	0.7667	30	0.8570	32	Regenerative	Zibo	0.4663	47	0.6398	42	0.8269	42
Mature	Yangquan	0.6680	18	0.8078	28	0.8186	47								

**Table A2:** Main paths of inefficient decomposition of high-quality input-output allocation and improvement of efficiency in mining cities.

Category	City	TGRI	GMI	MTI	Improved Group Technology	Improve Management Level	Category	City	TGRI	GMI	MTI	Improved Group Technology	Improve Management Level
Mature	Anshun	15.1510%	45.1210%	60.2720%			Mature	Yichun	22.8149%	54.9300%	77.7449%	△	△
Mature	Baise	6.6974%	65.9910%	72.6884%		△	Mature	Yunfu	6.2855%	57.9230%	64.2085%		
Mature	Baoji	12.4792%	58.4510%	70.9302%			Mature	Yuncheng	8.9069%	68.4610%	77.3679%		△
Mature	Baoshan	21.2818%	46.3340%	67.6158%	△	△	Mature	Zhangjiakou	6.7936%	63.4420%	70.2356%		
Mature	Benxi	11.2415%	44.1470%	55.3885%			Mature	Changzhi	6.6868%	60.3840%	67.0708%		
Mature	Bozhou	17.6493%	20.5880%	38.2373%	△	△	Mature	Chongqing	12.7538%	58.8760%	71.6298%		
Mature	Chenzhou	15.4206%	44.8850%	60.3056%			Mature	Zigong	10.5843%	37.0620%	47.6463%		
Mature	Chengde	9.9744%	46.1660%	56.1404%			Growth	Erdos	17.8593%	6.2010%	24.0603%	△	△
Mature	Chizhou	27.6574%	50.9620%	78.6194%	△	△	Growth	Hezhou	13.3986%	35.4500%	48.8486%		
Mature	Chifeng	10.3710%	40.3280%	50.6990%			Growth	Hulunbeier	14.6677%	16.6420%	31.3097%	△	△
Mature	Chuzhou	11.8856%	41.5510%	53.4366%			Growth	Liupanshui	8.0563%	57.4530%	65.5093%		
Mature	Dazhou	11.0205%	41.8350%	52.8555%			Growth	Nanchong	1.9239%	8.2120%	10.1359%	△	△
Mature	Daqing	19.2546%	4.6190%	23.8736%	△	△	Growth	Shuozhou	12.5621%	2.6120%	15.1741%	△	△
Mature	Datong	23.5753%	57.0930%	80.6683%	△	△	Growth	Songyuan	23.3950%	6.2250%	29.6200%		
Mature	Dongying	15.2171%	7.9760%	23.1931%			Growth	Xianyang	10.4266%	40.3240%	50.7506%		
Mature	Ezhou	16.6618%	36.8080%	53.4698%			Growth	Yulin	3.3498%	12.7660%	16.1158%	△	△
Mature	Ganzhou	18.8174%	53.8710%	72.6884%			Decay	Baishan	11.9393%	13.5770%	25.5163%	△	△
Mature	Guang’an	12.8356%	8.3300%	21.1656%	△	△	Decay	Fushun	9.4038%	12.6770%	22.0808%	△	△
Mature	Guangyuan	13.5062%	34.1740%	47.6802%			Decay	Fuxin	11.6737%	27.0350%	38.7087%		
Mature	Handan	25.5493%	50.5290%	76.0783%	△	△	Decay	Hegang	9.7875%	9.0720%	18.8595%	△	△
Mature	Hechi	1.2450%	60.4380%	61.6830%			Decay	Huaibei	12.5642%	37.5940%	50.1582%		
Mature	Hebi	18.6894%	54.3270%	73.0164%			Decay	Huang	12.7652%	30.0190%	42.7842%		
Mature	Heihe	5.6874%	13.0630%	18.7504%	△	△	Decay	Jiaozuo	3.0476%	15.7660%	18.8136%		
Mature	Hengyang	1.2827%	55.8590%	57.1417%			Decay	Jingdezhen	13.0055%	33.5540%	46.5595%		
Mature	Huzhou	4.9125%	54.6980%	59.6105%			Decay	Liaoyuan	16.1688%	3.3370%	19.5058%		
Mature	Huainan	13.7679%	55.6460%	69.4139%			Decay	Luzhou	14.2279%	26.3720%	40.5999%	△	△
Mature	Jixi	6.2764%	18.9300%	25.2064%	△	△	Decay	Pingxiang	8.3512%	29.9100%	38.2612%		
Mature	Jilin	15.9454%	48.7120%	64.6574%			Decay	Puyang	0.9080%	8.3740%	9.2820%	△	△
Mature	Jining	14.1464%	52.2550%	66.4014%			Decay	Qitaihe	11.9816%	7.9330%	19.9146%	△	△
Mature	Jincheng	25.3164%	47.5210%	72.8374%	△	△	Decay	Shaoguan	2.4614%	45.0590%	47.5204%		
Mature	Laiwu	22.6440%	36.4360%	59.0800%	△	△	Decay	Tongling	19.3833%	6.7660%	26.1493%		
Mature	Lincang	14.9152%	43.7970%	58.7122%			Decay	Wuhai	4.6080%	2.1240%	6.7320%	△	△
Mature	Linfen	13.0564%	57.4850%	70.5414%			Decay	Xinyu	15.0056%	14.9920%	29.9976%	△	△
Mature	Longyan	4.8077%	43.0440%	47.8517%			Decay	Yichun	19.4123%	13.3380%	32.7503%	△	△
Mature	Loudi	20.9915%	56.0940%	77.0855%	△	△	Decay	Zaozhuang	11.0289%	18.4310%	29.4599%	△	△
Mature	Mudanjiang	7.8532%	38.8190%	46.6722%			Regenerative	Anshan	8.1150%	37.8210%	45.9360%		
Mature	Nanping	17.7634%	47.6840%	65.4474%			Regenerative	Baotou	12.0467%	6.5930%	18.6397%	△	△
Mature	Panzhihua	12.3170%	34.6650%	46.9820%			Regenerative	Huludao	23.7931%	51.7010%	75.4941%	△	△
Mature	Pingdingshan	4.3632%	57.8110%	62.1742%			Regenerative	Lijiang	18.3619%	5.9230%	24.2849%	△	△
Mature	Qujing	13.3212%	56.3870%	69.7082%			Regenerative	Linyi	7.1122%	45.3200%	52.4322%		
Mature	Sanmenxia	5.8291%	47.9450%	53.7741%			Regenerative	Luoyang	9.0703%	37.4120%	46.4823%		
Mature	Sanming	9.8013%	57.4910%	67.2923%			Regenerative	Ma’anshan	11.3359%	40.4970%	51.8329%		
Mature	Shaoyang	11.0470%	17.8970%	28.9440%	△	△	Regenerative	Nanyang	7.8475%	40.0450%	47.8925%		
Mature	Suzhou	17.7324%	26.7560%	44.4884%	△	△	Regenerative	Panjin	11.7662%	5.3860%	17.1522%	△	△
Mature	Taian	9.8022%	31.7820%	41.5842%			Regenerative	Suqian	10.1492%	28.9970%	39.1462%		
Mature	Weinan	6.9498%	62.4680%	69.4178%			Regenerative	Tangshan	10.3168%	39.6040%	49.9208%		
Mature	Xingtai	8.5563%	62.5200%	71.0763%			Regenerative	Tonghua	22.0770%	53.7810%	75.8580%	△	△
Mature	Xuancheng	6.1716%	43.7260%	49.8976%			Regenerative	Xuzhou	22.3566%	45.5990%	67.9556%	△	△
Mature	Ya’an	10.9657%	23.3330%	34.2987%			Regenerative	Zibo	11.0727%	36.0220%	47.0947%		

△ reflects main path of improvement of efficiency in mining cities.

## References

[1] Zhang N., Zhou P., Choi Y. (2013). Energy efficiency, CO_2_ emission performance and technology gaps in fossil fuel electricity generation in Korea: A meta-frontier non-radial directional distance function analysis. Energy Policy.

[2] Yan D., Kong Y., Ye B., Shi Y., Zeng X. (2019). Spatial variation of energy efficiency based on a Super -Slack-Based Measure: Evidence from 104 resource-based cities. J. Clean. Prod..

[3] Jing Z., Wang J. (2020). Sustainable development evaluation of the society–energy– environment in a resource-based city of China: A complex network approach. J. Clean. Prod..

[4] Liu X., Meng X. (2018). Evaluation and empirical research on the energy efficiency of 20 mining cities in Eastern and Central China. Int. J. Min. Sci. Technol..

[5] Ruan F., Yan L., Wang D. (2020). The complexity for the resource-based cities in China on creating sustainable development. Cities.

[6] Song M., Zhao X., Shang Y. (2020). The impact of low-carbon city construction on ecological efficiency: Empirical evidence from quasi-natural experiments. Resour. Conserv. Recycl..

[7] Chen W., Chen W., Ning S., Liu E., Zhou X., Wang Y., Zhao M. (2019). Exploring the industrial land use efficiency of China’s resource-based cities. Cities.

[8] Deng W. (2019). Evaluating Transformation Efficiency of Resource-based Coastal Cities: An AHP and DEA Based Analysis. J. Coast. Res..

[9] Hu Y., Yan T., Chen F. (2020). Energy and Environment Performance of Resource-Based Cities in China: A Non-Parametric Approach for Estimating Hyperbolic Distance Function. Int. J. Environ. Res. Public Health.

[10] Xiao H., Wang D., Qi Y., Shao S., Zhou Y., Shan Y. (2021). The governance-production nexus of eco- efficiency in Chinese resource-based cities: A two-stage network DEA approach. Energy Econ..

[11] Bui T.-D., Tsai F.M., Tseng M.-L., Wu K.-J., Chiu A.S. (2020). Effective municipal solid waste management capability under uncertainty in Vietnam: Utilizing economic efficiency and technology to foster social mobilization and environmental integrity. J. Clean. Prod..

[12] Yuan J., Bian Z., Yan Q., Pan Y. (2019). Spatio-Temporal Distributions of the Land Use Efficiency Coupling Coordination Degree in Mining Cities of Western China. Sustainability.

[13] Zhang H., Shen L., Zhong S., Elshkaki A. (2020). Economic Structure Transformation and Low- Carbon Development in Energy-Rich Cities: The Case of the Contiguous Area of Shanxi and Shaanxi Provinces, and Inner Mongolia Autonomous Region of China. Sustainability.

[14] Yu Y., Huang J., Zhang N. (2019). Modeling the eco-efficiency of Chinese prefecture-level cities with regional heterogeneities: A comparative perspective. Ecol. Model..

[15] Yin Q., Wang Y., Wan K., Wang D. (2020). Evaluation of green transformation efficiency in Chinese mineral resource-based cities based on a three-stage DEA method. Sustainability.

[16] Battese G.E., Rao D.S.P. (2002). Technology gap, efficiency, and a stochastic metafrontier function. Int. J. Bus. Econ..

[17] O’Donnell C.J., Rao D.S., Battese G.E. (2008). Metafrontier frameworks for the study of firm-level efficiencies and technology ratios. Empir. Econ..

[18] Liu W.B., Zhang D.Q., Meng W., Li X.X., Xu F. (2011). A study of DEA models without explicit inputs. Omega.

[19] Wang D., Li S., Sueyoshi T. (2014). DEA environmental assessment on US Industrial sectors: Investment for improvement in operational and environmental performance to attain corporate sustainability. Energy Econ..

[20] He J.H., He C.H., Sedighi H.M. (2021). Evans model for dynamic economics revised. AIMS Math..

[21] Liu Y. (2020). Analysis of the Efficiency of Environmental Regulation on the Transformation of the Resource-based Cities. IOP Conf. Ser. Earth Environ. Sci..

[22] Peng J., Wen L., Fu L., Yi M. (2020). Total factor productivity of cultivated land use in China under environmental constraints: Temporal and spatial variations and their influencing factors. Environ. Sci. Pollut. Res..

[23] Baumol W.J. (1986). Productivity Growth, Convergence, and Welfare: What the Long-Run Data Show. Am. Econ. Rev..

[24] Anselin L. (1988). Lagrange multiplier test diagnostics for spatial dependence and spatial heterogeneity. Geogr. Anal..

[25] Qunwei W., Peng Z., Dequn Z. (2014). Heterogeneity of production technology, carbon dioxide emission and performance lose: An international comparison based on meta-frontier. Sci. Res. Manag..

[26] Chiu Y.H., Huang K.Y., Chang T.H., Lin T.Y. (2021). Efficiency assessment of coal mine use and land restoration: Considering climate change and income differences. Resour. Policy.

[27] Xiao W., Zhang H.Y., Zhang J.Y. (2014). GIS-based Analysis of LS Factor under Coal Mining Subsidence Impacts in Sandy Region. J. Eng. Sci. Technol. Rev..

